# A Differential Clinical Diagnostic Challenge of a Recurrent, Oral Fibrosarcoma Resembling a Periapical Lesion of Endodontic Origin

**DOI:** 10.7759/cureus.49836

**Published:** 2023-12-02

**Authors:** Vasileios Zisis, Athanasios Poulopoulos, Ioannis Fotopoulos, Theodoros Lillis, Nikolaos Dabarakis, Eleftherios Anagnostou, Dimitrios Andreadis

**Affiliations:** 1 Oral Medicine/Pathology, School of Dentistry, Faculty of Health Sciences, Aristotle University of Thessaloniki, Thessaloniki, GRC; 2 Dentoalveolar Surgery, Implantology and Oral Radiology, School of Dentistry, Faculty of Health Sciences, Aristotle University of Thessaloniki, Thessaloniki, GRC; 3 Oral Medicine/ Pathology, School of Dentistry, Faculty of Health Sciences, Aristotle University of Thessaloniki, Thessaloniki, GRC

**Keywords:** recurrent sarcoma, immunohistochemistry staining, fibrosarcoma, head and neck neoplasms, oral diseases

## Abstract

A fibrosarcoma is a neoplastic growth originating from malignant, fibroblast-like mesenchymal cells. This malignant tumor shows an increased tendency for expansion and recurrence and a propensity to metastasize, especially to the lungs. Despite their rarity, fibrosarcomas have the potential to manifest in any anatomical location. An oncologist referred their patient due to reported mandibular discomfort, ache, and swelling. The biopsy revealed a fibrosarcoma resembling a periapical lesion of endodontic origin. The timely intervention and the collaboration among different but complementary medical and dental specialties ensure that the patient may enjoy a prolonged life expectancy as symptom-free as possible.

## Introduction

A fibrosarcoma is an infrequent neoplasm of mesenchymal origin but not only. The precise origins of fibrosarcomas remain uncertain, as there is limited understanding of their etiology. Fibrosarcomas predominantly manifest in the soft tissues, although cases of intraosseous lesions have also been documented [1]. Approximately 5-10% of fibrosarcomas originate in the head and neck region, specifically in the nose, paranasal sinuses, and the skin and subcutaneous tissue in these areas [2]. The primary sites affected by oral fibrosarcomas are the jaws. The clinical manifestation of oral fibrosarcomas includes symptoms such as pain, swelling, paraesthesia, tooth mobility, and ulceration of the mucosal layer [3]. The clinical manifestations of fibrosarcomas exhibit variability contingent upon factors such as the tumor size, anatomical site, and extent of metastasis. Common symptoms associated with this condition may encompass sensations of discomfort, inflammation, or the development of open sores [4-6]. A fibrosarcoma originating in the oral cavity typically presents as a morphologically lobulated, sessile, painless, and non-hemorrhagic submucosal mass with normal pigmentation. In contrast, aggressive cases of fibrosarcomas exhibit a propensity for swift growth and present as a hemorrhagic mass. In terms of clinical manifestation, the differential diagnosis includes pyogenic granuloma, peripheral giant cell granuloma, peripheral ossifying fibroma, etc. Even in cases where lesions do not exhibit surface ulceration or rapid growth, they may still manifest destruction of the underlying muscle and bone [4-6]. In this case study, we present an uncommon manifestation of a recurrent fibrosarcoma in a patient, characterized by the initial presence of a painless gingival mass and the subsequent, both intraosseous and gingival, recurrence in a different quadrant of the oral cavity resembling a periapical lesion of endodontic origin.

## Case presentation

**Figure 1:**
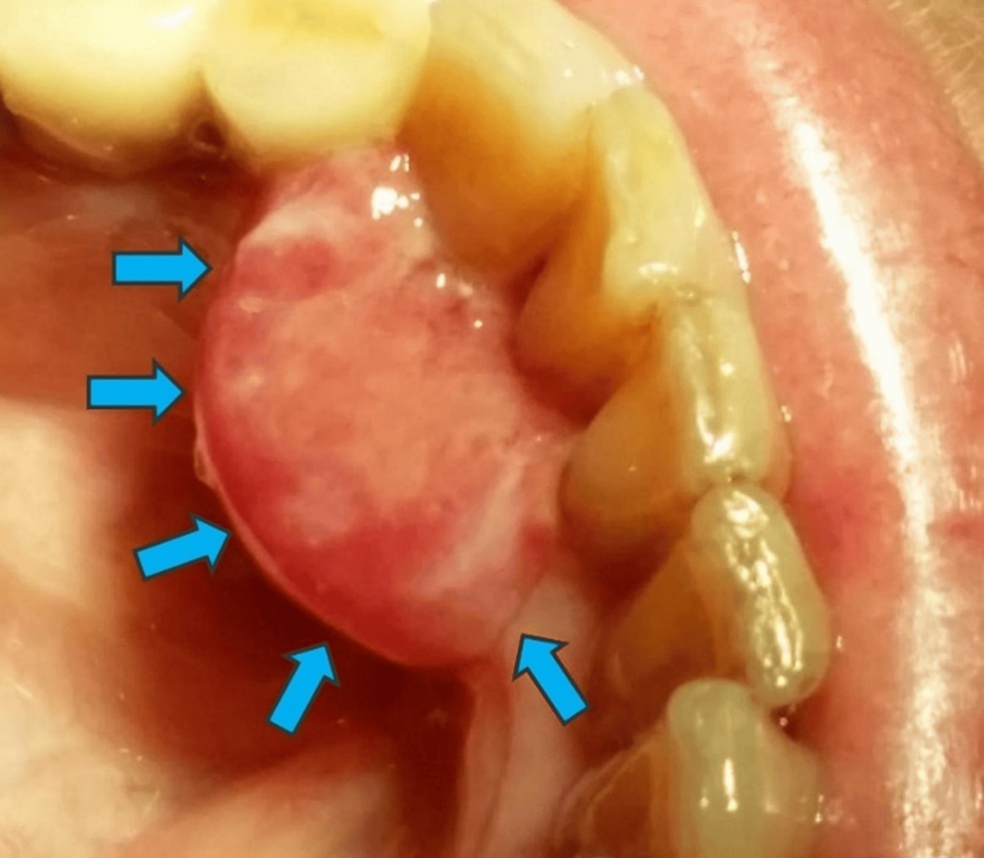
The gingival mass may be noticed on the lingual gingiva of teeth #31-33 (blue arrows).

A 60-year-old female patient presented to the Department of Oral Medicine/Pathology, School of Dentistry, Aristotle University of Thessaloniki, Thessaloniki, Greece, following a referral from her oncologist. The patient reported experiencing discomfort, ache, and swelling in the right half of her mandible. The patient provided a description of the pain as being continuous, without any explicit reference to certain factors that either alleviate or exacerbate it. Prior to the commencement of the examination, the patient duly furnished written consent after being adequately apprised of the relevant details and implications. The aforementioned form of written consent received approval from the School of Dentistry at Aristotle University of Thessaloniki, since it adhered to the principles outlined in the Helsinki Declaration on research and ethical considerations for patients. The patient reported that six months before the initial gingival mass, a dermatologist removed from her scalp a macule, which was deemed to be benign and was therefore not submitted to histopathological examination. The dental history revealed the removal of a malignant tumor of mesenchymal origin two years ago, located on the lingual gingiva of teeth #31-33 and the respective figure was retrieved (Figure 1).

**Figure 2:**
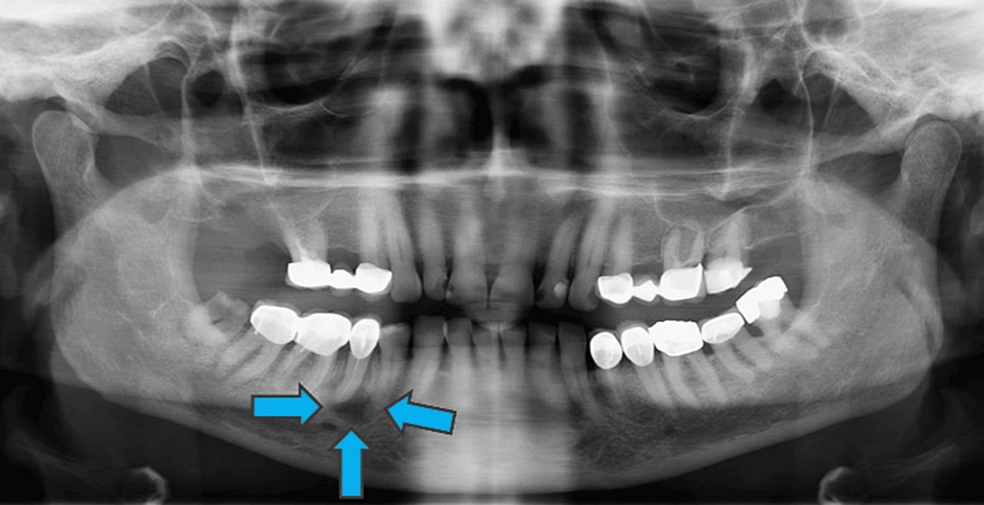
OPG examination revealing the intraosseous lesion (blue arrows). OPG: Orthopantomogram

In addition to surgical removal, the patient received chemotherapy. After one year, the patient was referred for a second time due to a swelling in the gingiva of the left part of the mandible. The clinical examination and orthopantomogram (OPG) examination revealed a lesion extended to the periapical area of tooth #45 (Figure 2).

**Figure 3:**
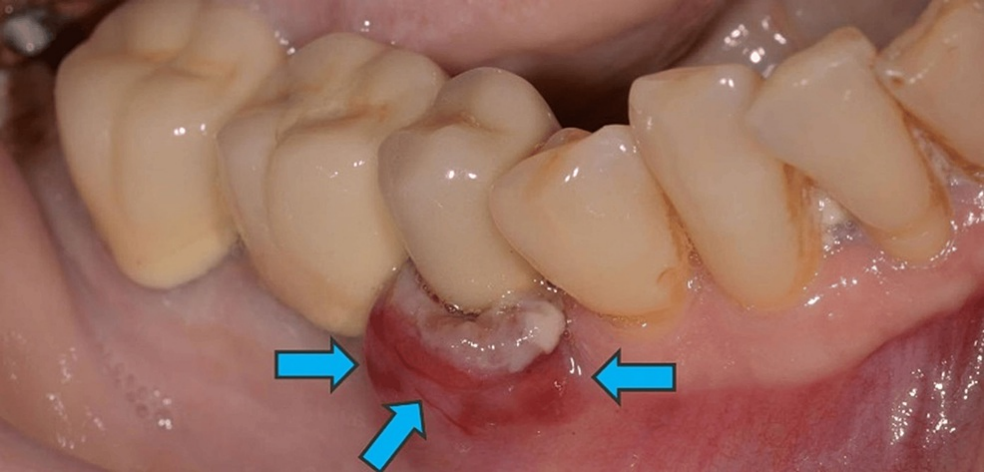
Prior to the surgical intervention, a picture was taken of the area under investigation (blue arrows).

The OPG also revealed an incomplete endodontic treatment of tooth #45. The lesion was sessile and was characterized by a soft consistency and a reddish discoloration (Figure 3).

**Figure 4:**
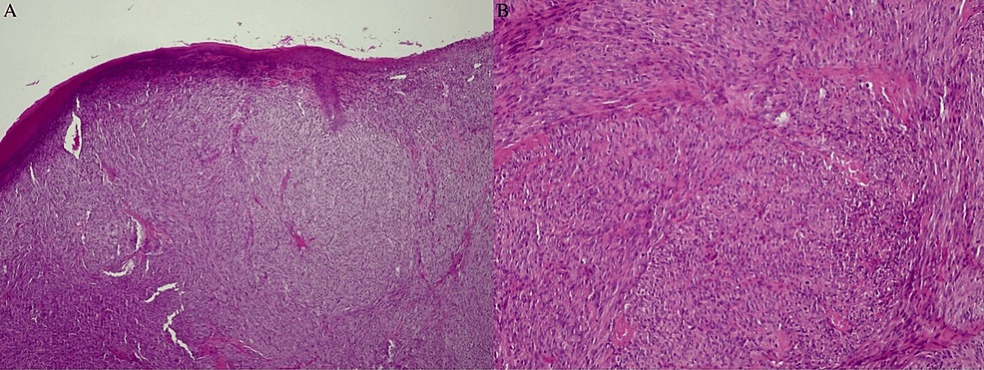
A lesion of mesenchymal origin with sarcomatoid features of moderate cellularity, with eosinophilic cytoplasm, elongated fusiform nuclei, mild nuclear atypia, and abundant mitoses (A X10), (B X20).

The patient was subsequently referred for endodontic treatment. However, after six weeks, the lesion persisted and the tooth was finally extracted. The site of the biopsy included the region of tooth #45 and in particular, the buccal soft tissue and the intrabony lesion as well (as shown in the OPG examination). The type of biopsy performed was a full excisional biopsy (including the affected oral mucosa and the intrabony lesion). The lesion was then preserved in formaldehyde and submitted to a full histopathological examination. The microscopic appearance of the tissue specimen showed a lesion of mesenchymal origin with sarcomatoid features of moderate cellularity, with eosinophilic cytoplasm, elongated fusiform nuclei, mild nuclear atypia, and abundant mitoses (Figure 4).

**Figure 5:**
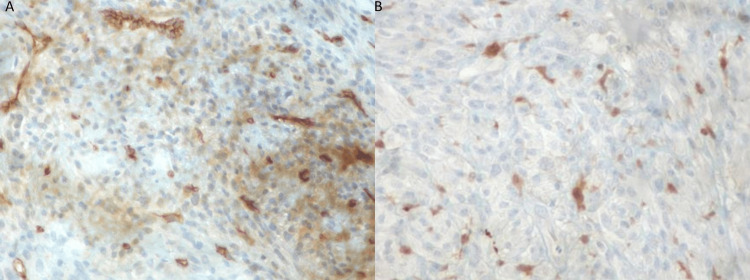
A: Fibrosarcoma cells exhibit mild, diffuse positivity for CD34 (immunoperoxidase staining X400). B: Scattered neoplastic cells are strongly positive for S100 protein (immunoperoxidase staining X400).

Therefore, the histopathological examination showed a mesenchymal cancer with features indicative of a fibrosarcoma. The immunohistochemical staining included the examination of CD34(+) (Figure 5A), S100(+) (scanty positivity) (Figure 5B), h-caldesmon(-), Melan-A(-), desmin(-), and broad-spectrum cytokeratins AE1-AE3(-). The patient was referred to her oncologist for further treatment.

## Discussion

A fibrosarcoma is an aggressive neoplasm originating from fibroblast-like mesenchymal cells. The aforementioned sarcoma variant primarily manifests in the vicinity of osseous structures or within soft tissue. Malignancies originating from fibroblasts are infrequently observed in the oral and oropharyngeal area [7]. However, it is noteworthy that a fibrosarcomas is the predominant form of mesenchymal cancer in this region, accounting for over 50% of all sarcomas. The precise etiology of a fibrosarcoma remains incompletely elucidated; nevertheless, it has been suggested that genetic modifications may contribute to its pathogenesis since a considerable age range exists, and a significant number of patients affected by this condition are under the age of 20 [6,8]. Infantile or congenital fibrosarcoma represents the prevailing soft tissue sarcoma observed in children who are less than one year old [6,7]. However, fibrosarcoma occurring in extracutaneous sites typically manifest in individuals within the age range of 25 to 79 years. The tumor exhibits its highest occurrence rate within the age range of 55-69 years [6,7]. Sex predilection is not reported. Factors that may contribute to the development of fibrosarcomas in the oral cavity are the presence of scar tissue, exposure to elevated temperatures, and certain diseases such as Paget's disease and osteomyelitis [6-8]. The involvement of buccal mucosa, tongue, and alveolus collectively accounts for over 50% of the reported cases [7]. The differential diagnosis for fibrosarcomas includes reactive fibromatosis, pseudosarcomatous fasciitis, fibroblastic osteogenic sarcoma, cellular alveolar sarcoma, and generally, tumors containing spindle-shaped cells [9]. The absence of muscle tissue markers (such as in our case) confirms the diagnosis of fibrosarcomas. Accurate diagnosis necessitates a thorough examination of multiple sections, utilization of special stains, and immunohistochemical analysis [4, 7]. Low-grade fibrosarcomas may be composed of CD34-positive cells [10]. Positive S100 is indicative of low-grade myofibroblastic sarcoma [11]. However, the muscle involvement is excluded since both h-caldesmon and desmin were negative. H-caldesmon is considered a specific marker for tumors with smooth muscle differentiation [12]. Desmin is a sensitive and specific marker for muscle differentiation [13]. Melan-A (negative) is a melanocytic differentiation marker [14]. Broad-spectrum cytokeratins AE1-AE3 (also negative) are indicative of epithelial involvement [15]. Another case of fibrosarcoma located in the gingiva was reported in the literature [3]. Metastatic fibrosarcomas are exceptionally uncommon and are rarely reported in the literature [16]. Radical surgery is considered the preferred treatment for fibrosarcomas. Radiation therapy and chemotherapy may be employed as therapeutic modalities in cases where surgical intervention is not feasible or as a means of providing palliative care. The prognosis of the tumor is contingent upon factors such as histological grade, tumor size, and the implementation of appropriate surgical intervention with the achievement of disease-free margins. The prognosis for this particular disease is characterized by a low five-year survival rate, typically falling within the range of 20% to 35% [7,17]. The radiolucency of the periapical lesion of tooth #45 resembled a lesion of endodontic origin, thus perplexing the differential diagnostic process. After the failure of the revision of the endodontic treatment, the extraction of the tooth constituted the next step to alleviate the symptoms of the patient and the biopsy that was carried out solved the differential diagnostic dilemma. Such a case is not reported in the literature.

## Conclusions

A fibrosarcoma is a neoplastic growth originating from fibroblast-like mesenchymal cells, characterized by malignant nature, tendency for rapid expansion, recurrence, and propensity to metastasize. In case of involvement of the maxillofacial area, and the oral cavity in particular, diagnostic dilemmas may arise due to the resemblance to other pathological entities. A stepwise approach ensures the best clinical outcome, even in complicated cases. The fibrosarcoma that is presented constitutes such an example, due to the overlap of clinical and radiographical appearance with a typical periapical endodontic lesion. Cases such as the one presented illustrate the crucial importance of submitting tissue specimens of every lesion which are removed for histopathological investigation. Finally, the timely intervention and the collaboration among different but complementary medical and dental specialties ensure that the patient may enjoy a prolonged life expectancy as symptom-free as possible.
